# Fatigue Life Assessment of High-Strength Stainless Steels via Small Punch Testing

**DOI:** 10.3390/ma19112365

**Published:** 2026-06-02

**Authors:** Ran Li, Wenbo Li, Wenshu Wei, Rongming Chen, Mengyu Wu, Hao Liu, Jian Ye, Jianfeng Li, Yuehua Lai, Tianze Cao, Fengcai Liu

**Affiliations:** 1 Graduate School, China Coal Research Institute, Beijing 100013, China; 2 State Key Laboratory of Intelligent Coal Mining and Strata Control, Beijing 100013, China; 3 Beijing Tianma Intelligent Control Technology Co., Ltd., Beijing 101399, China; 4 College of Mechanical and Electronic Engineering, Shandong University of Science and Technology, Qingdao 266590, China

**Keywords:** small punch fatigue tests, scanning electron microscopy, finite element, X17CrNi15-2, 15-5PH, PH13-8Mo

## Abstract

Small punch fatigue tests (SPFTs) were conducted on three high-strength stainless steels: X17CrNi15-2, 15-5PH, and PH13-8Mo. The SPFT valley displacement-versus-SPFT life curves for the three stainless steels exhibited three different stages. Power-law relationships were obtained to characterize the maximum forces with SPFT lives for different stainless steels. The fracture mechanisms of the tested SPFT specimens were characterized via scanning electron microscopy, which was dependent on the materials and applied loads. Finite element analyses were performed to obtain the equivalent local stresses and strains. A simplified critical plane-strain energy density (SED) criterion was used for the SPFT life assessment by correlating the FE-obtained strain energy density with the SPFT life. The SED values for the SPFT life were in the following order: PH13-8Mo > 15-5PH > X17CrNi15-2.

## 1. Introduction

Many components are exposed to cyclic loading and tend to fail because of fatigue. Therefore, it is critical to carry out a fatigue life assessment of these components to evaluate their remaining lives and provide suggestions on repairs or replacements. Small punch tests (SPTs) only require small amounts of material to be extracted from in-service components for the life assessments of those components, which does not destroy their structural integrity. Hence, it is vital to apply SPTs to the fatigue life assessments of in-service components or structures.

SPTs were initially designed for the characterization of different material properties, such as tensile properties [1,2,3], fracture toughness [4,5], and creep properties [6,7]. In recent years, some studies have attempted to develop small punch fatigue testing techniques for fatigue life prediction. Villarraga et al. [8] conducted small punch fatigue tests (SPFTs) on oxidized and unoxidized ultrahigh-molecular-weight polyethylene. Tasdighi et al. [9] proposed an SPT-based method for predicting the axial fatigue life of 304 stainless steel that employed the Palmgren–Miner model to estimate the cyclic load history. Lancaster et al. [10] performed a series of innovative small punch fatigue tests on the titanium alloy Ti-6Al-4V and correlated them with uniaxial fatigue data by using finite element analyses. Lewis et al. [11] investigated the fatigue performance of Ti-6Al-4V and C263 additively manufactured materials in comparison with other variants. Prakash and Arunkumar [12] established a novel cyclic small punch testing procedure by which to assess the degradation of the material properties of copper, brass, and SS-304. They also proposed an empirical equation correlating the fatigue damage and hysteresis energy during cyclic SPT. Wang et al. [13] evaluated the defect-related fatigue performance of additively manufactured GH4169 and used various machine learning algorithms to predict the small punch fatigue life.

Despite the above progress in small punch fatigue testing, most existing studies mainly focus on conventional carbon steels, titanium alloys and common austenitic stainless steels. Limited attention has been paid to the fatigue behavior and life evaluation of high-strength martensitic and precipitation-hardening stainless steels under small punch cyclic loading. Moreover, previous investigations rarely combined the Chaboche combined hardening constitutive model with finite element simulation to characterize the multiaxial stress–strain evolution of SPFT specimens. In addition, a unified strain energy density criterion for tensile-dominated small punch fatigue failure still lacks systematic simplification and calibration for high-strength stainless steel systems.

To fill these research gaps, this study carries out systematic small punch fatigue tests on three typical high-strength stainless steels, namely X17CrNi15-2, 15-5PH, and PH13-8Mo. The fatigue life is correlated with applied maximum load via a power-law function, and the fracture failure mechanisms are revealed by SEM observation. Meanwhile, the Chaboche combined hardening model is adopted in finite element analysis to obtain the local equivalent stress and strain distribution of SPFT specimens. Furthermore, a simplified critical plane-strain energy density criterion is established to evaluate the small punch fatigue life of the three stainless steels. The novelty of this work lies in the quantitative characterization of fatigue performance, fracture mechanism and strain energy density life correlation for the three high-strength stainless steels, which provides a reference for the fatigue life assessment and engineering application of such materials based on small punch testing technology.

In this study, small punch fatigue tests were carried out for three different stainless steels, namely, X17CrNi15-2, 15-5PH, and PH13-8Mo, to evaluate the fatigue properties of the three stainless steels from a small volume of materials. The SPFT lifetimes were initially correlated with the maximum forces by using a power relationship for each stainless steel. Scanning electron microscopy (SEM) was performed to determine the fracture modes of the SPFT specimens. Numerical investigations were performed using a finite element model in conjunction with the Chaboche combined hardening model to obtain the stress and strain distributions of SPFT specimens under different loads. Strain energy densities obtained from finite element analyses were used to correlate the SPFT lives.

## 2. Small Punch Testing

### 2.1. Materials and Test Specimens

**Figure 1:**
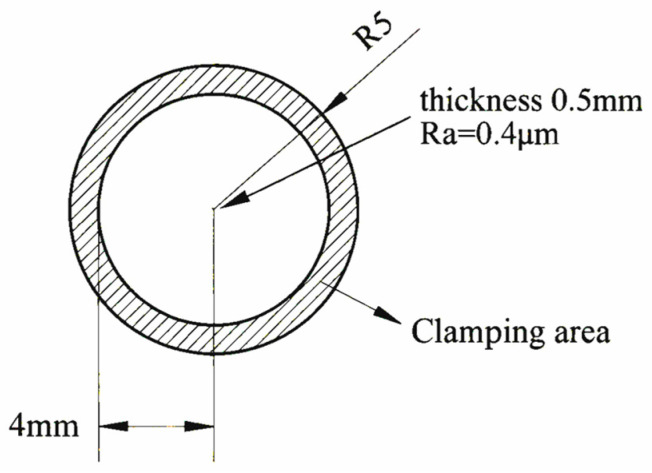
The specimen for the small punch test.

The tensile properties of the martensitic stainless steel X17CrNi15-2, as well as two martensitic precipitation-hardening stainless steels, namely, 15-5PH and PH13-8Mo, were investigated in our previous study [14]. In this study, SPFTs were carried out on the three stainless steels. The chemical properties of stainless steel are listed in Table 1. The SPFT specimens were fabricated into small discs with a diameter of 10 mm, a thickness of 0.5 mm and an arithmetic mean roughness of *R_a_* = 0.4 µm, in accordance with GB/T 29459.2-2012 [15]. The specific diagram is shown in Figure 1, which presents the specimen for the small punch test.

### 2.2. Test Apparatus and Procedure

**Figure 2:**
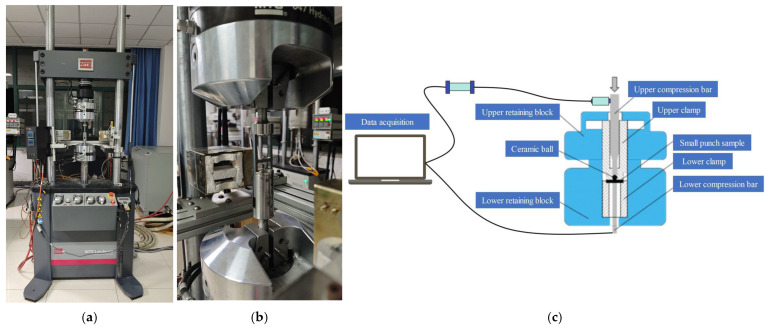
(**a**) Test rig and (**b**) test fixture; (**c**) schematic diagram of SPFT.

**Figure 3:**
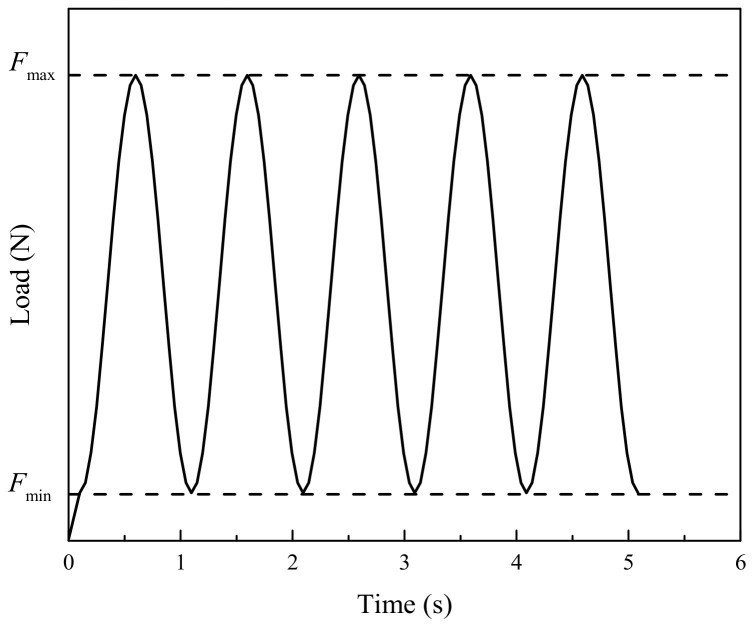
In-phase sinusoidal load profile of SPFT.

The test rig consisted of an MTS Landmark 370 hydraulic servo testing machine (MTS Systems Corporation, Eden Prairie, MN, USA) and an SPFT fixture, as illustrated in Figure 2. As shown in Figure 2b, the fixture consists of a lower and upper die, lower and upper punch rod, clamping screw, clamping cover, lower and upper pressing block, and locking nut. The load-controlled fatigue method was employed, using in-phase sinusoidal load profiles with a loading ratio of *R* = 0.1 and a frequency of 1 Hz, as illustrated in Figure 3. A preload of 20 N was applied to ensure microcontact between the test punch and SPFT specimens.

### 2.3. SPFT Results

**Figure 4:**
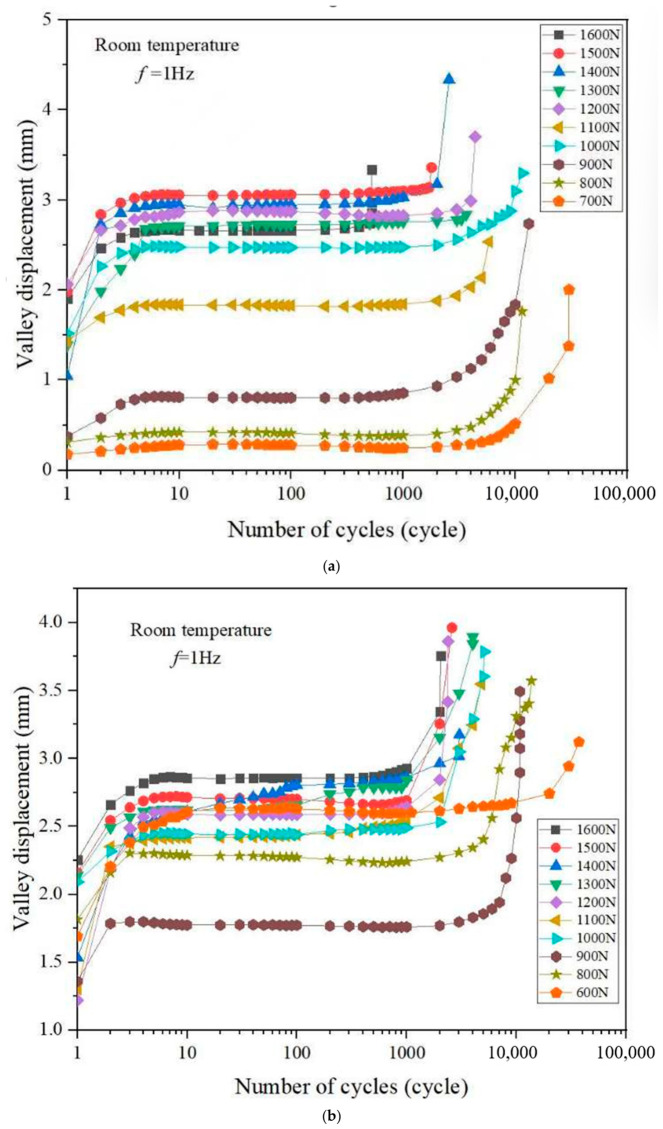

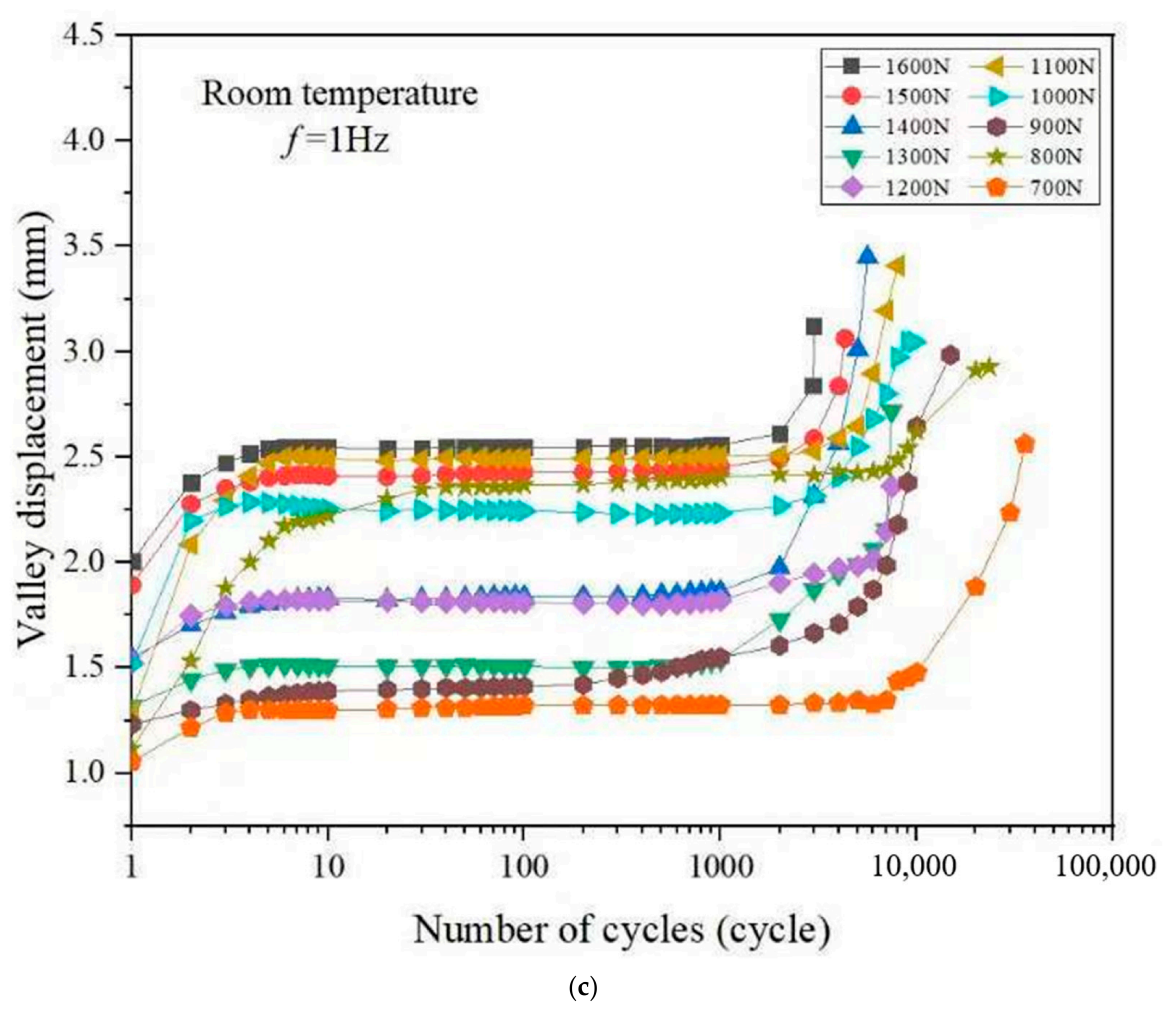
The SPFT valley displacement-versus-SPFT life curves for three stainless steels: (**a**) X17CrNi15-2; (**b**) 15-5PH; (**c**) PH13-8Mo.

Figure 4 shows the SPFT valley displacement-versus-SPFT life curves for X17CrNi15-2, 15-5PH and PH13-8Mo, respectively. Valley displacement refers to the minimum displacement value recorded in each loading–unloading cycle during the small punch fatigue test (SPFT). Figure 4 shows that regardless of the value of the maximum force and type of stainless steel, the valley displacement increases gradually at the early stage of the SPFT, tends to remain stable, and then grows suddenly and rapidly at the final stage. The characteristics of the different stages of the SPFT valley displacement versus SPFT life curves are in good agreement with the results of previous studies [16,17,18]. The horizontal axis of Figure 3 represents the number of load cycles N. In accordance with the standard criterion for small punch fatigue testing, the SPFT fatigue life N_f_ is defined as the number of cycles when the central displacement of the specimen decreases by 10% relative to the maximum displacement, or at the moment of specimen fracture, whichever occurs first.

It can be observed that the valley displacement curves under Figure 4b 600 N and Figure 4c 800 N show obvious abnormal trends compared with other load levels. This anomalous behavior is mainly attributed to the moderate load amplitude at 600 N and 800 N, which induces intermittent local contact separation and repeated micro-plastic deformation inside the specimen. Different from the stable deformation state under low load and severe plastic accumulation under high load, such an intermediate load leads to irregular fluctuation of valley displacement with the increase in cycle number, presenting a deviation from the conventional variation tendency.

**Figure 5:**
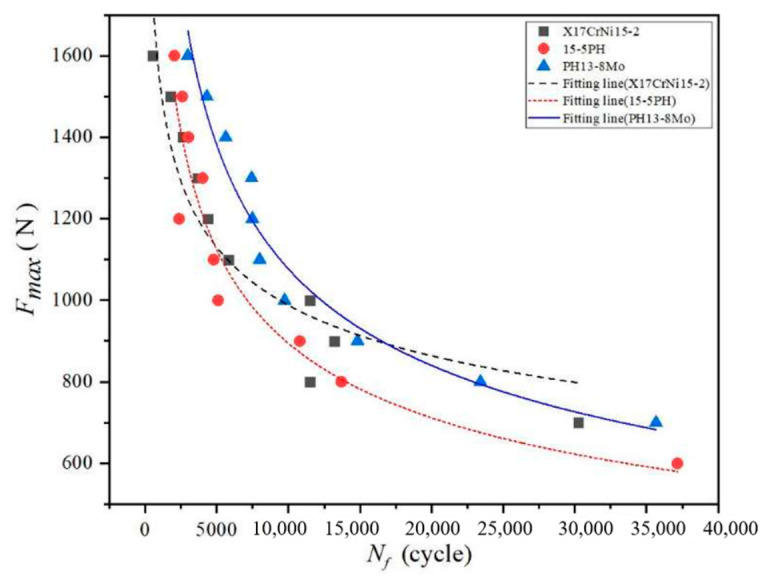
The maximum forces versus the SPFT lives for three stainless steels.

Figure 5 shows the maximum forces versus the SPFT lifetimes for X17CrNi15-2, 15-5PH, and PH13-8Mo. The SPFT life decreases with an increasing maximum force. When the maximum force was higher than 1200 N, the variation in SPFT life with the maximum force was small. However, with a decrease in the maximum force, the variation in the SPFT life increases with the maximum force. The power-law relationship for each stainless steel sample can be obtained by correlating the maximum forces with the SPFT life, and it is shown in Equation (1). Table 2 summarizes the values of parameters *A* and *n* for the three stainless steels.

(1) $$F_{\max}=A{N_{f}}^{n}$$

### 2.4. SEM Morphology Analysis

Scanning Electron Microscopy (SEM) (MTS Systems Corporation, Eden Prairie, MN, USA) was employed to observe the fracture morphology, surface damage characteristics and crack propagation behavior of SPFT specimens. Before observation, the fractured specimens were cut, ultrasonically cleaned and dried to remove surface contaminants. The fracture surfaces were then examined by SEM at different magnifications. The microscopic features of the fatigue source zone, crack propagation zone and final fracture zone were analyzed to reveal the fatigue failure mechanism of the material under cyclic loading.

**Figure 6:**
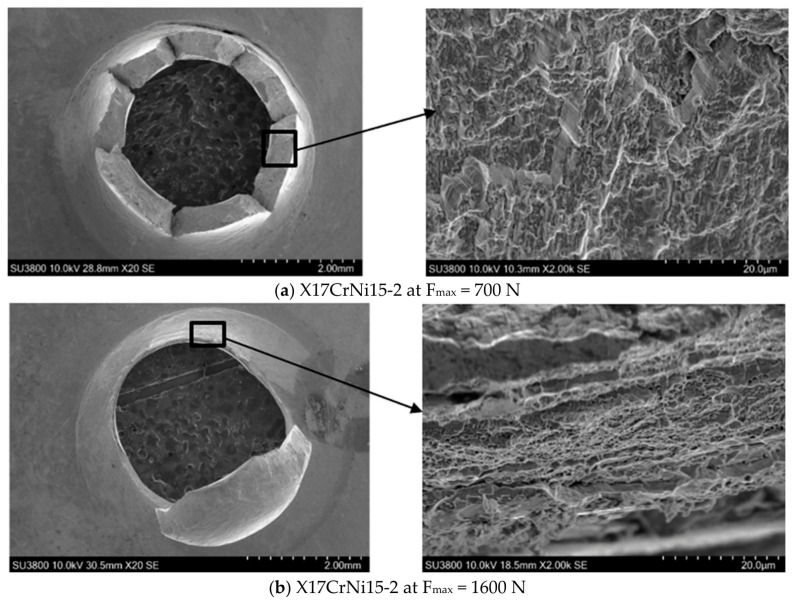
SEM fractographs on the ruptured surface of the SPFT specimens for X17CrNi15-2 at minimum and maximum F_max_.

**Figure 7:**
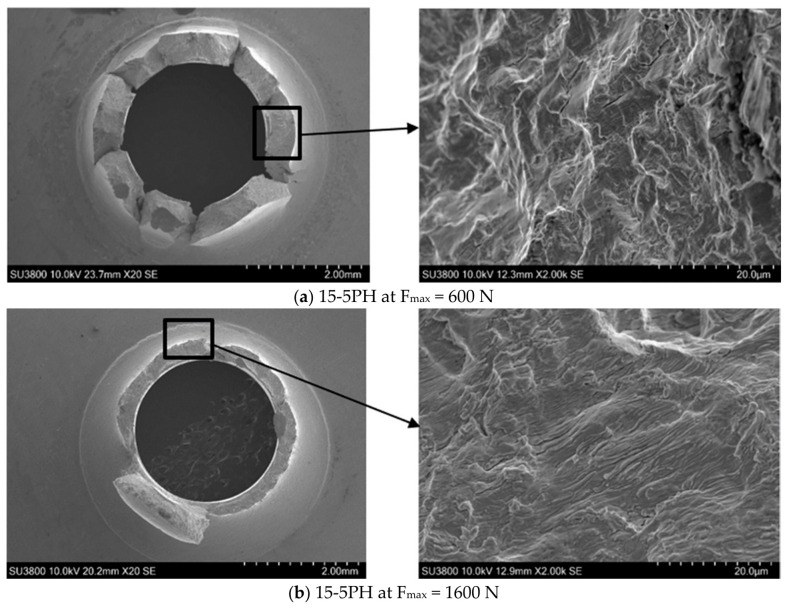
SEM fractographs on the ruptured surface of the SPFT specimens for 15-5PH at minimum and maximum F_max_.

**Figure 8:**
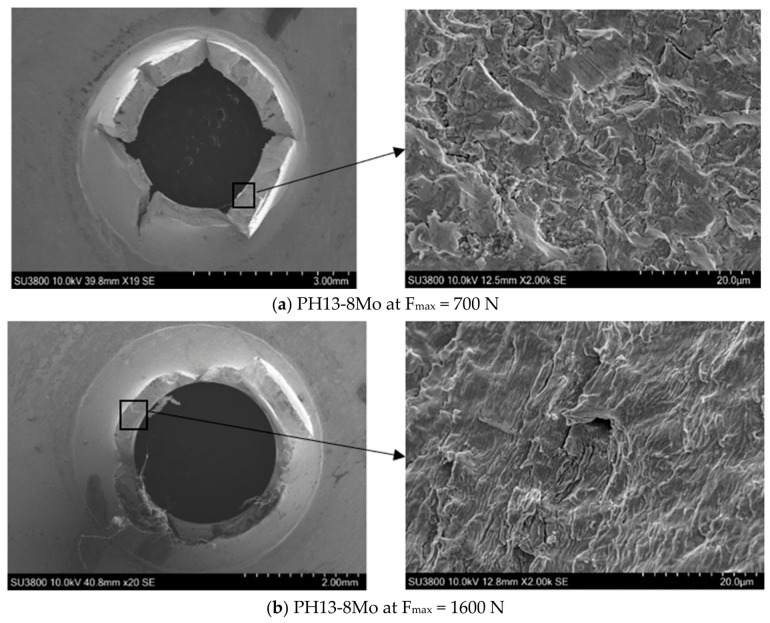
SEM fractographs on the ruptured surface of the SPFT specimens for PH13-8Mo at minimum and maximum F_max_.

The SEM images of the fracture surfaces of the tested SPFT specimens at the maximum and minimum values of F_max_ were obtained for the three stainless steels. SEM micrographs of a lower magnification at the minimum values of F_max_ for the three stainless steels, as shown in Figure 6a, Figure 7a and Figure 8a, indicate that, except for the part near the center of the specimen that cracks along the circumferential direction, the rest of the cracks propagate radially. However, as shown in Figure 6b, Figure 7b and Figure 8b, the fracture morphologies of the three stainless steels at the maximum values of F_max_ were characterized by circumferential cracks and no obvious radial cracks. In the SEM micrographs of a higher magnification, dimple fracture for X17CrNi15-2, and fatigue striations for 15-5PH and PH13-8Mo can be observed at the maximum values of F_max_. For the other SEM micrographs at higher magnifications, internal microcracks were observed for the three stainless steels at the minimum values of F_max_.

## 3. Finite Element Analyses of Small Punch Fatigue Tests

### 3.1. Chaboche Combined Hardening Model

#### 3.1.1. Nonlinear Kinematic Hardening Rule of the Chaboche Model

The nonlinear kinematic hardening model employs a combination of different back stresses to describe the changes in the position of the yield surface in space, as shown in Equation (2):

(2) $$\alpha =\sum_{k=1}^{3}\alpha_{k};{\dot{\alpha }}_{k}=C_{k}\frac{1}{\sigma^{0}}(\sigma -\alpha ){\dot{\bar{\varepsilon }}}^{pl}-\gamma_{k}{\dot{\bar{\varepsilon }}}^{pl}$$

where $\alpha_{k}$ is the *k*th backstress, α is the total backstress, $C_{k}$ and $\gamma_{k}$ are model parameters corresponding to the *k*th backstress, and $\sigma^{0}$ is the initial yield stress radius.

#### 3.1.2. Isotropic Hardening Rule of the Chaboche Model

The isotropic hardening rule of the Chaboche model describes the variation in the yield surface size with the equivalent plastic strain ${\dot{\bar{\varepsilon }}}^{pl}$:

(3) $${\dot{\sigma }}^{0}=Q_{\infty }(b\cdot e^{-{\bar{\varepsilon }}^{pl}}){\dot{\bar{\varepsilon }}}^{pl}$$

(4) $$\sigma^{0}={\left.\sigma \right|}_{0}+Q_{\infty }(1-e^{-b{\bar{\varepsilon }}^{pl}})$$

where ${\left.\sigma \right|}_{0}$ is the yield stress at zero plastic strain, $Q_{\infty }$ is the maximum range of variation in the size of the yield surface, and *b* is the rate at which the yield surface size changes with plastic strain.

In the Chaboche combined hardening model [19], *b* is determined based on the shape and geometric size of the standard small punch test fixture, and $\sigma^{0}$ is obtained from the standard small punch tests. These data come from a previous study [14]. The $C_{k}$ and $\gamma_{k}$ terms are qualitatively determined from a large number of small punch fatigue tests for different materials using iterative finite element analyses. The Young’s modulus, denoted by *E*, and Poisson’s ratio, denoted by *v*, were obtained from standard small punch tests. These data also come from a previous study [14]. The material constants in the Chaboche combined hardening model, as well as the *E* and *v* values for the three stainless steels, are listed in Table 3.

## 4. Finite Element Analyses

**Figure 9:**
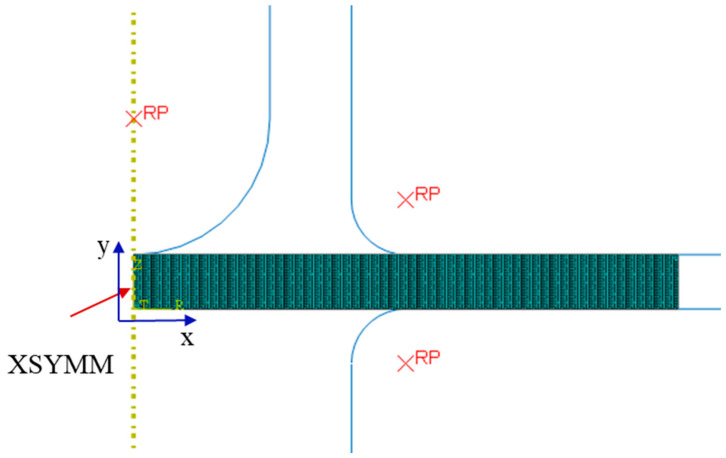
Finite element model for SPFTs.

A two-dimensional axisymmetric finite element (FE) model was generated for the SPC test system using the commercial code Abaqus 2022 [20]. The FE model is shown in Figure 9 and consists of a small disc, with a thickness of 0.5 mm and a radius of 5 mm, a punch indenter with a radius of 1.25 mm, and the upper and lower dies with rounded corners of 0.5 mm. The receiving hole had a radius of 2.0 mm. The punch, upper die, and lower die in the FE model were defined as rigid bodies, and reference points were used for the three rigid bodies such that the motion could be substituted for the motion of the rigid bodies. In addition, four-noded bilinear asymmetrical (CAX4R) elements were used to mesh the SPFT specimens. The SPFT specimen was meshed with a total of 6250 solid elements, and each element has a uniform volume of 0.001 mm^3^. Mesh refinement was adopted in the key stress and crack propagation regions to ensure calculation accuracy, while a relatively uniform element size was arranged in the remaining areas to balance computational efficiency and simulation precision. A uniform friction coefficient of 0.3 was used for the punch specimen, upper-die specimen, and lower-die specimen contacts.

**Figure 10:**
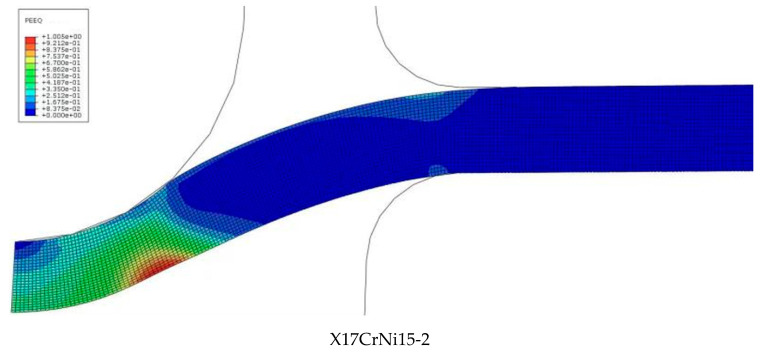

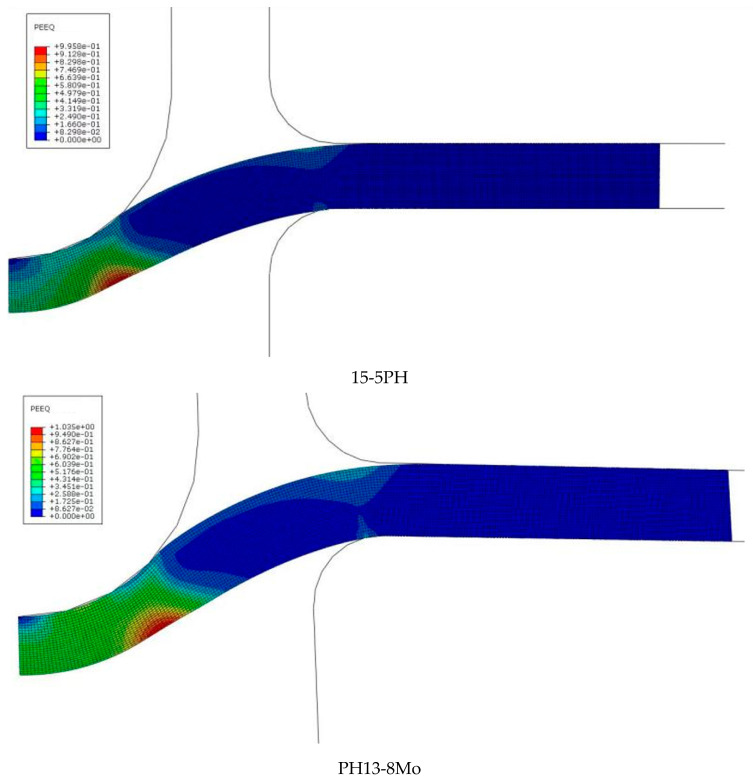
Typical FE equivalent plastic strain distributions of 15-5 PH, PH 13-8Mo and Ni2 SPFT specimens at F_max_ = 800 N.

Figure 10 shows the typical FE-equivalent plastic strain distributions of the X17CrNi15-2, 15-5 PH, and PH 13-8Mo SPFT specimens at F_max_ = 800 N. The maximum equivalent plastic strain occurred on the lower surfaces of all three stainless steel SPFT specimens. It can be seen from the equivalent strain distribution in Figure 10 that the maximum equivalent strain occurs at the edge contact region of the specimen, which is basically consistent with the fatigue crack initiation sites observed from the SEM fracture morphologies in Figure 6, Figure 7 and Figure 8. This indicates that the region with high strain concentration is prone to local plastic deformation and stress concentration, thereby inducing fatigue crack initiation. The simulation results are well verified by the SEM microscopic experimental observations.

## 5. Discussion

The SPFT specimen experienced a complex stress state under multiaxial stress conditions. In this study, the fatigue lives of all three specimens were less than 10^−5^ cycles, categorized as low-cycle fatigue. Owing to the complexity of the small punch fatigue test, this study employed a finite element analysis to correlate the F_max_-N_f_ curve with the ε-N_f_ curve. The principle of the correlation is that the SPFT and uniaxial fatigue specimens have the same fatigue life when their energy parameters are equivalent. Cracks in small punch fatigue specimens typically initiate on the outer surfaces, with failure primarily occurring because of circumferential cracks caused by thin-film stretching. Consequently, it is believed that the SPFT lives of the three stainless steel specimens were primarily tensile-dominated, although shearing also played a role in the failure stages. To comprehensively consider the effects of normal stress, normal strain, shear stress, and shear strain on the fatigue life of small punch bars, the model of Chen et al. [21] was adopted for the multiaxial fatigue life prediction. The energy–life formula is

(5) $$\frac{\Delta \varepsilon_{1}\Delta \sigma_{1}}{4}+\frac{\Delta \gamma_{1}\Delta \tau_{1}}{4}=B\cdot {N_{f}}^{m}$$

In Equation (5), $\frac{\Delta \varepsilon_{1}}{2}$ and $\frac{\Delta \sigma_{1}}{2}$ are the maximum positive strain amplitude and positive stress amplitude on the critical plane, $\frac{\Delta \gamma_{1}}{2}$ and $\frac{\Delta \tau_{1}}{2}$ are the shear strain amplitude and shear stress amplitude on the critical plane, and *A* and *B* are material parameters.

For SPFTs, because failure is mainly caused by tension, shear stress and shear strain can be ignored. Hence, Equation (5) can be simplified as

(6) $$\frac{\Delta \varepsilon_{1}\Delta \sigma_{1}}{4}=B\cdot {N_{f}}^{m}$$

(7) $$W=\frac{\Delta \varepsilon_{1}\Delta \sigma_{1}}{4}$$

where *W* is the strain energy density (SED) of the tensile-dominated stress states of the SPFTs. The strain energy density–life (W–N) equation was derived using the SPFT results in conjunction with the numerical results obtained from the FE analyses.

The critical plane is the plane with the maximum positive strain amplitude during the fatigue cycle. The expression for the positive strain amplitude $\frac{\Delta \varepsilon_{1}}{2}$ on the critical plane is

(8) $$\frac{\Delta \varepsilon_{1}}{2}=\frac{1}{2}\left(\varepsilon_{x1}-\varepsilon_{x2}\right)$$

where $\varepsilon_{x1}$ and $\varepsilon_{x2}$ are the positive strains on the section under the maximum force F_max_ and minimum force F_min_, respectively.

The stress distribution of the SPFT specimen was extremely complicated, and it was necessary to determine the dangerous point as a reference point. In general, the larger the energy parameter, the smaller the SPFT life. Therefore, the dangerous point with the largest energy parameter was selected as the reference point. Crack initiation usually occurs on the lower surface of an SPFT specimen. Therefore, a lower-surface area should be compared to select the largest strain energy density. When the point of the SED is the largest, the point is the dangerous point and can be used as the reference point of the SPFT specimen for fatigue life prediction. Hence, the energy parameters of the stress and strain histories can be determined from the danger points under different loads. Table 4 lists the energy parameter sizes corresponding to different loads. Table 4 shows that the energy parameters increased with the load.

The strain energy density *W* for the tensile-dominated stress states of the SPFTs at the danger points can be determined from the FE stress and strain data under different forces. Table 4 shows the strain energy densities corresponding to the different forces, and the results indicate that the strain energy density increased with an increase in the force.

**Figure 11:**
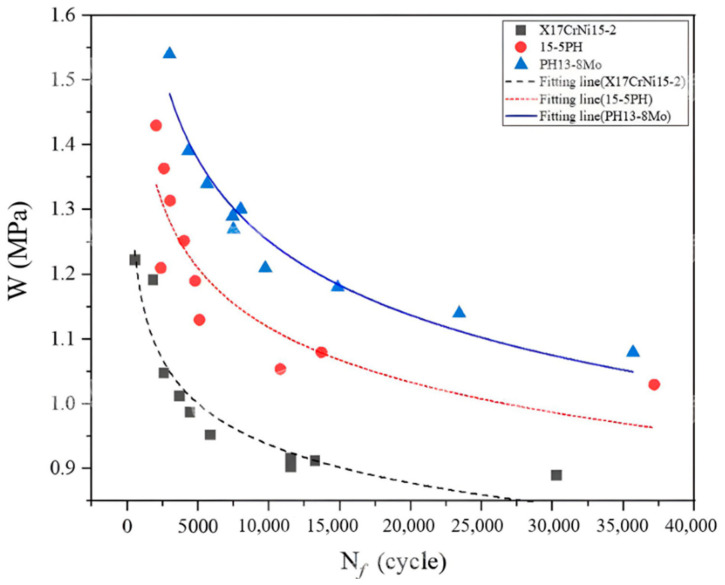
The strain energy density-versus-fatigue life relationships for the three different stainless steels.

The SPFT method enables the fatigue life evaluation of in-service components by extracting only a small amount of material. It is necessary to verify the consistency between SPFT results and those obtained by conventional fatigue testing methods. Figure 10 presents the comparative results of SPFT and traditional fatigue tests. Further quantitative analysis shows that the SPFT data are in good agreement with the conventional fatigue test results, which verifies the reliability and rationality of applying the SPFT method to the life assessment of in-service components. Figure 11 shows the strain energy density-versus-fatigue life relationships for the three different stainless steels. Figure 11 shows that the SPFT fatigue life decreases with increasing strain energy density for each stainless steel. For the same SPFT life, the value of the SED was in the order PH13-8Mo > 15-5PH > X17CrNi15-2. Equation (5) can be obtained for each stainless steel sample to evaluate its SPFT fatigue life. Table 5 shows the *B* and *m* values of Equation (5) for the three stainless steels derived in Figure 11.

The fatigue evolution behavior of the component is characterized by the variation in load with the number of cycles in the experiment, while the finite element simulation adopts the variation in strain energy with the number of cycles for analysis. In terms of physical essence, the internal force dissipation caused by external loading and the accumulation of strain energy belong to the same fatigue damage evolution process. Both can reflect the laws of stiffness degradation, plastic accumulation and fatigue deterioration of components under cyclic loading, with only different characterization parameters selected. To ensure the computational convergence and simulation accuracy of the finite element model, some model parameters are reasonably calibrated and appropriately modified, resulting in slight differences between the simulation parameters and the original experimental parameters. By comparing Figure 4 and Figure 10, it can be found that the overall evolution trend of the strain energy–cycle curve is highly consistent with that of the load–cycle curve, which proves that the simulation results can effectively verify the experimental laws, and there is good internal correlation and consistency between them.

## 6. Conclusions

In this study, to explore the fatigue behavior of three stainless steels (i.e., X17CrNi15-2, 15-5PH, and PH13-8Mo), small punch fatigue tests were conducted using disc-type specimens under different loadings. The conclusions are as follows.

- The SPFT valley displacement-versus-SPFT life curves show that the valley displacement increases gradually in the early stage of the SPFT, tends to remain stable, and then grows suddenly and rapidly in the final stage. Power-law relationships were obtained for the stainless steels by correlating the maximum forces with the SPFT lives.
- SEM analyses revealed that increasing the value of F_max_ eliminated radial cracks. SEM micrographs illustrated that dimple fractures for X17CrNi15-2 and fatigue striations for 15-5PH and PH13-8Mo were observed at the maximum values of F_max_, whereas internal microcracks were observed for the three stainless steels at the minimum values of F_max_.
- Finite element analyses were performed to obtain equivalent local stresses and strains. The FE results suggested that fatigue failure occurred on the lower surfaces of the three stainless steel SPFT specimens. The strain energy densities for the tensile-dominated stress states of the SPFTs were defined. The strain energy densities at the dangerous points can be determined from the FE stress and strain data under different forces. The correlation between the strain energy density and SPFT life was obtained by simplifying the model of Chen et al. The SPFT life increased with an increasing strain energy density. For the same fatigue life, PH13-8Mo had the largest strain energy density, and X17CrNi12 had the smallest strain energy density.
- Although the small punch test technique has been widely used to evaluate the mechanical properties and fatigue behavior of materials, this work is not merely a routine application of the mature method to three stainless steels. According to the actual service load and working-condition characteristics of the plunger pump power end components, a targeted small punch cyclic fatigue test scheme is designed. Meanwhile, finite element simulation is introduced to establish a coupled analysis framework combining experimental measurement and numerical simulation, from the aspects of valley displacement evolution, equivalent strain distribution, strain energy variation and crack initiation location.
- By comparing the stiffness degradation, deformation response and microscopic damage mechanism of the three stainless steels under cyclic loading, the differentiated fatigue evolution mechanisms of different materials are revealed. The obtained results provide experimental basis and theoretical support for material selection, fatigue performance evaluation and anti-fatigue optimization of pump structural components. The present work shows certain characteristics and novelty in condition-oriented test design, multi-material comparative study, and correlation mechanism interpretation between simulation and experiment.

## Figures and Tables

**Table 1:** Chemical compositions of test specimens (wt%) [14].

Materials	C	P	Si	Mo	Cr	Mn	Fe	Al	Ni	Nb	S	Cu	Ta
X17CrNi15-2	0.18	0.023	0.37		16.46	0.62	Bal.		2.34		0.0048		
15-5PH	0.056	0.028	0.6		14.32	0.58	Bal.		4.21	0.33	0.0028	3.15	0.0006
PH13-8Mo	0.021	0.0076	0.43	2.15	12.57	0.02	Bal.	1.11	8.36		0.0009		

**Table 2:** Summary of the values of *A* and *n* for the three stainless steels.

Materials	A	*n*
X17CrNi15-2	7086.994	−0.219
15-5PH	11,199.935	−0.378
PH13-8Mo	21,863.433	−0.387

**Table 3:** The material constants in the Chaboche combined hardening model for the three stainless steels.

Materials	C_1_/MPa	γ_1_	C_2_/MPa	γ_2_	C_3_/MPa	γ_3_	${\left.\sigma \right|}_{0}$/MPa	Q∞/MPa	b	E/GPa	υ
X17CrNi15-2	349,202	9171	42,849	597	2985	3.5	707.35	45	13.2	190	0.3
15-5PH	350,000	9000	42,849	597	2985	3.5	1067.6	45	13.2	197	0.3
PH13-8Mo	355,000	9100	42,000	597	3000	3.5	1174.3	45	13.2	200	0.3

**Table 4:** The energy parameter sizes corresponding to different loads.

Maximum Load/N	600	700	800	900	1000	1100	1200	1300	1400	1500	1600
W_X17CrNi15-2_		0.891	0.903	0.913	0.917	0.953	0.988	1.013	1.049	1.192	1.223
W_15-5PH_	1.03		1.08	1.054	1.13	1.19	1.21	1.252	1.314	1.363	1.43
W_PH13-8Mo_		1.08	1.14	1.18	1.21	1.301	1.27	1.29	1.34	1.391	1.54

**Table 5:** The parameters in the power-law relationship for correlating the strain energy density-versus-fatigue life relationships.

Materials	B	m
X17CrNi15-2	2.437	−0.931
15-5PH	3.784	−0.136
PH13-8Mo	4.957	−0.150

## Data Availability

The original contributions presented in this study are included in the article. Further inquiries can be directed to the corresponding author.

## References

[1] Bruchhausen M., Holmstrom S., Simonovski I., Austin T., Lapetite J.M., Ripplinger S., De Haan F. (2016). Recent developments in small punch testing: Tensile properties and DBTT. Theor. Appl. Fract. Mech..

[2] Haroush S., Priel E., Moreno D., Busiba A., Silverman I., Turgeman A., Shneck R., Geldstein Y. (2015). Evaluation of the mechanical properties of SS-316L thin foils by small punch testing and finite element analysis. Mater. Des..

[3] Song M., Gurao N.P., Qin W., Szpunar J.A., Guan K.S. (2015). Deciphering Deviation in Mechanical Properties of Differently Processed AISI 316L Austenitic Stainless Steel Using Small Punch Test. Mater. Sci. Eng. A.

[4] Foulds J., Viswanathan R. (2001). Determination of the toughness of in-service steam turbine disks using small punch testing. J. Mater. Eng. Perform..

[5] Lucon E., Benzing J.T., Derimow N., Hrabe N. (2021). Small punch testing to estimate the tensile and fracture properties of additively manufactured Ti-6Al-4V. J. Mater. Eng. Perform..

[6] Hyde T.H., Hyde C.J., Sun W. (2013). Theoretical basis and practical aspects of small specimen creep testing. J. Strain Anal. Eng. Des..

[7] Li R., Hyde T.H., Sun W., Dogan B. (2011). Modelling and data interpretation of small punch creep testing. Proceedings of the ASME 2011 Pressure Vessels and Piping Conference, Baltimore, MD, USA, 17–21 July 2011.

[8] Villarraga M.L., Edidin A.A., Herr M., Kurtz S.M. (2004). Multiaxial fatigue behavior of oxidized and unoxidized UHMWPE during cyclic small punch testing at body temperature. J. ASTM Int..

[9] Tasdighi E., Nobakhti H., Soltani N. (2016). Application of small punch test in predicting the axial fatigue life of 304 stainless steel sheets. Exp. Tech..

[10] Lancaster R.J., Jeffs S.P., Illsley H.W., Argyrakis C., Hurst R.C., Baxter G.J. (2019). Development of a novel methodology to study fatigue properties using the small punch test. Mater. Sci. Eng. A.

[11] Lewis D., Lancaster R., Jeffs S., Illsley H., Davies S., Baxter G. (2019). Characterising the fatigue performance of additive materials using the small punch test. Mater. Sci. Eng..

[12] Prakash R.V., Arunkumar S., Sokolov M.A., Lucon E. (2014). Evaluation of damage in materials due to fatigue cycling through static and cyclic small punch testing. Small Specimen Test Techniques: 6th Volume; STP 1576.

[13] Wang X., Xu L., Zhao L., Han Y. (2023). Evaluation of defect-related fatigue performance of additive manufacturing GH4169 via small punch test. Theor. Appl. Fract. Mech..

[14] Li R., Wei W., Chen R., Wu M., Lai Y. (2024). Evaluating the tensile properties of high-strength stainless steels using small punch testing. Sci. Prog..

[15] (2012). Small Punch Test Methods of Metallic Materials for In-Service Pressure Equipments—Part 1: General Requirements.

[16] Zhao L., Wang X., Xu L., Han Y., Jing H. (2021). Fatigue performance of Hastelloy X at elevated temperature via small punch fatigue test. Theor. Appl. Fract. Mech..

[17] Prakash R.V. (2017). Study of Fatigue Properties of Materials through Cyclic Automated Ball Indentation and Cyclic Small Punch Test Methods. Key Eng. Mater..

[18] Lancaster R.J., Illsley H., Hurst R., Jeffs S., Baxter G. (2017). A Novel Approach to Small Punch Fatigue Testing. Key Eng. Mater..

[19] Chaboche J.L. (2008). A review of some plasticity and viscoplasticity constitutive theories. Int. J. Plast..

[20] (2022). Abaqus 2022 Online Documentation. Dassault Systèmes. https://www.gmbinder.com/share/-OFvBLKQ6mhprbv-kBVn.

[21] Chen X., Xu S., Huang D. (2010). A critical plane-strain energy density criterion for multiaxial low-cycle fatigue life under non-proportional loading. Fatigue Fract. Eng. Mater. Struct..

